# Supplementary material for: Differentiation Induction of Mesenchymal Stem Cells by a Au Delivery Platform
Source: Cells. 2023 Jul 19;12(14):1893. doi: 10.3390/cells12141893 (PMC10378595; doi:10.3390/cells12141893)
Supplement: Supplementary file 1 [file cells-12-01893-s001.zip › cells-2365470-supplementary.pdf]

Differentiation Capacities Induction Accompanied with a Gold Nanoparticle Delivery Platform in Mesenchymal Stem Cells

Supplementary data

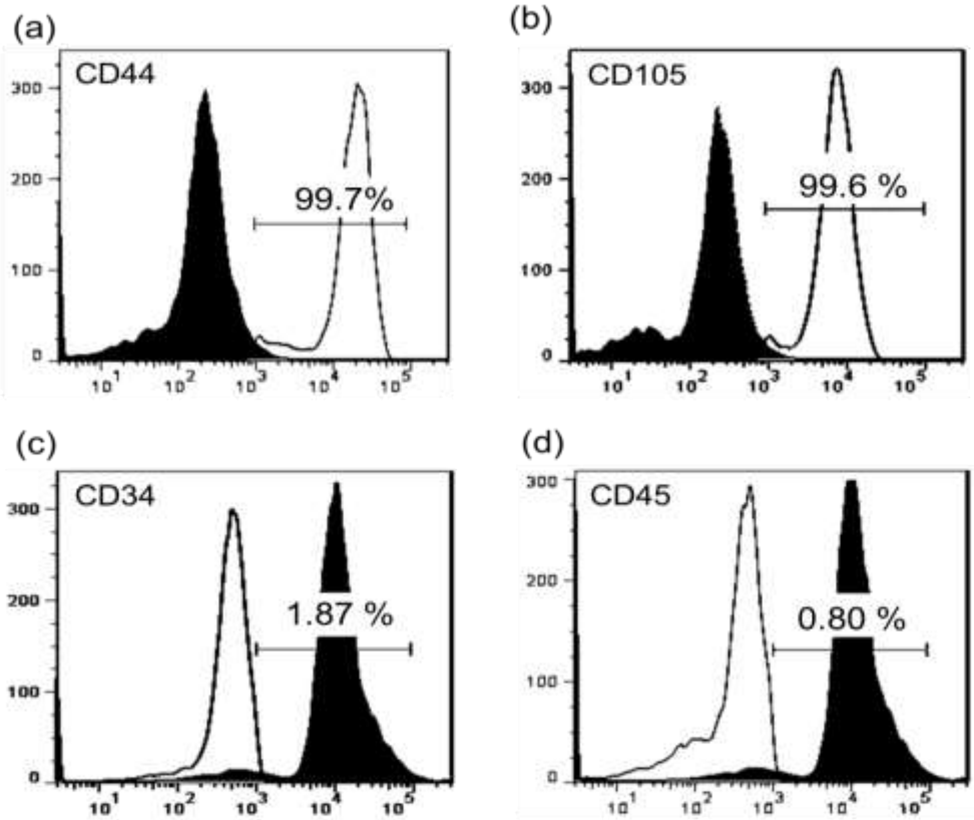

Figure S1. Phenotype identification of Wharton's jelly MSCs. The surface specific markers expression in cells were detected by flow cytometry. The expression of (a) CD44 and (b) CD105 positive markers were analyzed as 99.7% and 99.6 %. And the expression of (c) CD34 and (d) CD45 negative markers was 1.87% and 0.80% in cells, respectively. The above data represents of three independent experiments.

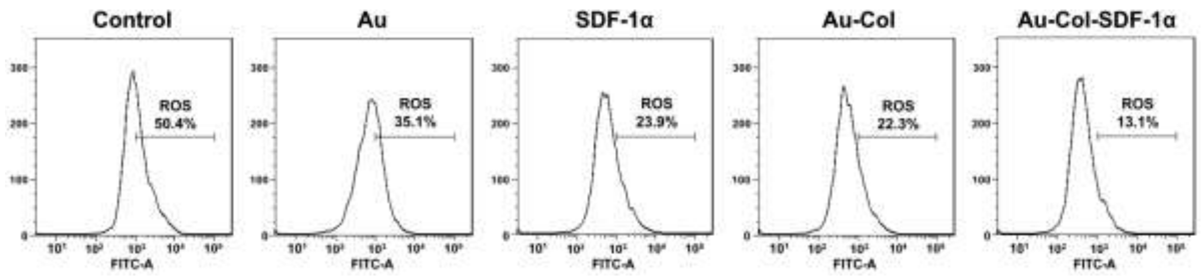

Figure S2. The FACS histograms of ROS fluorescein positive cells at 48 hours. The results are shown as one of three independent experiments.

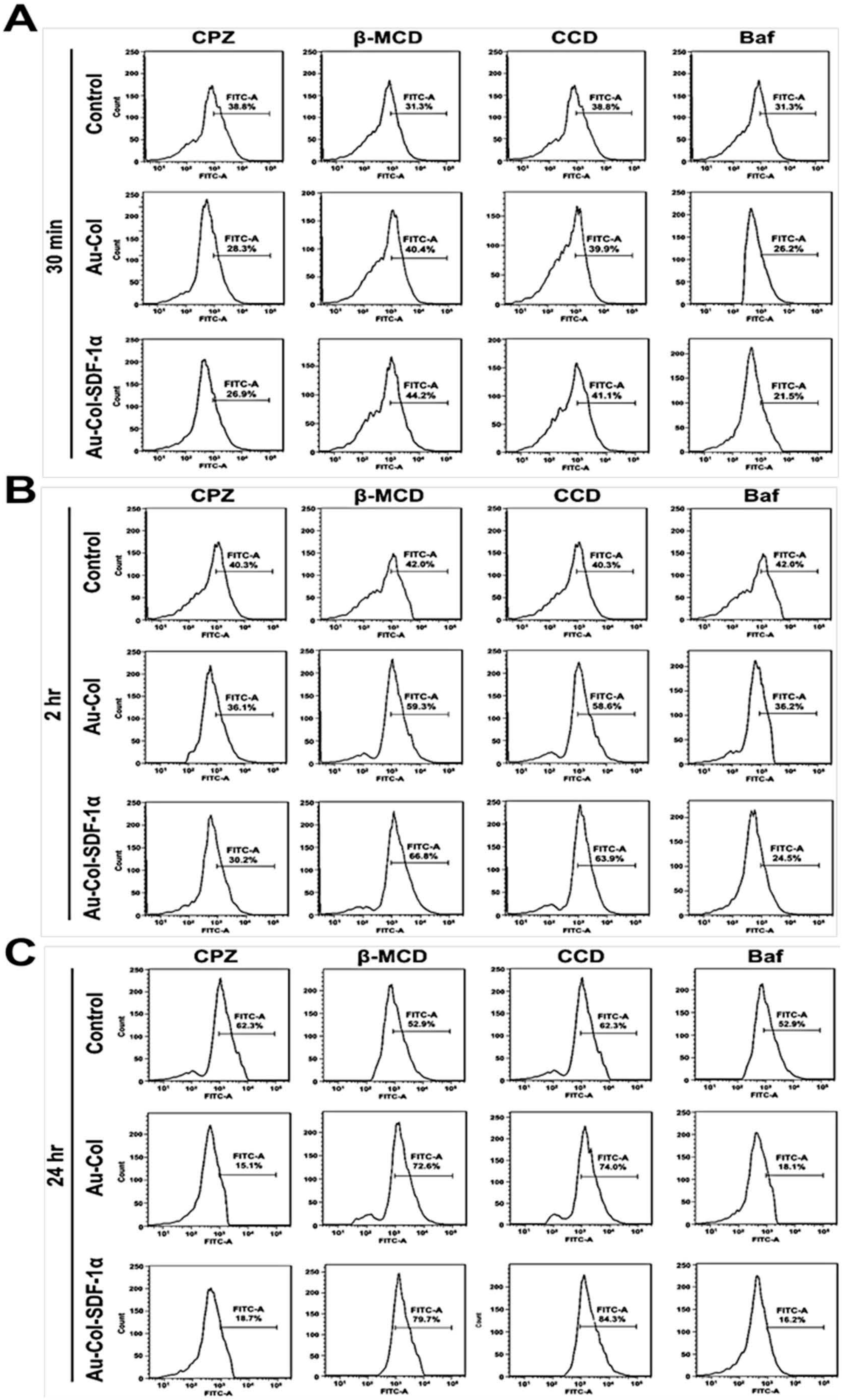

Figure S3. The FACS histograms of MSC uptake efficiency after being treated with four kinds of specific inhibitors are shown. Both Au-Col and Au-Col-SDF-1α treatments were investigated at (A) 30 minutes, (B) 2 hours and (C) 24 hours. The histograms were displayed as one of three independent experiments.

Table S1. CD31 gene expression levels in MSC.

CD31 expression	Relative fold
Control	1.00
Au	1.02
SDF-1α	0.94 *
Au-Col	0.96
Au-Col-SDF-1α	1.10 *

Note: * $p < 0.05$: compared to the control.

Table S2. vWF gene expression levels in MSC.

vWF expression	Relative fold
Control	1.00
Au	0.77
SDF-1α	0.76
Au-Col	0.92
Au-Col-SDF-1α	1.14 **

Note: ** $p < 0.01$: compared to the control.

Table S3. Nestin gene expression levels in MSC.

Nestin expression	Relative fold
Control	1.00
Au	0.85
SDF-1α	0.96
Au-Col	1.09
Au-Col-SDF-1α	1.19 *

Note: * $p < 0.05$: compared to the control.

Table S4. GFAP gene expression levels in MSC.

GFAP expression	Relative fold
Control	1.00
Au	0.93
SDF-1α	0.96
Au-Col	0.92
Au-Col-SDF-1α	0.95

Note: No significant difference between treatments.

Table S5. β-Tubulin gene expression levels in MSC.

β-Tubulin expression	Relative fold
Control	1.00
Au	0.62 ***
SDF-1α	0.79 ***
Au-Col	1.10
Au-Col-SDF-1α	1.19 *

Note: * $p < 0.05$, *** $p < 0.001$: compared to the control.

Table S6. Runx-2 gene expression levels in MSC.

Runx-2 expression	Relative fold
Control	1.00
Au	0.99
SDF-1α	1.22
Au-Col	1.30 **
Au-Col-SDF-1α	1.50 **

Note: ** $p < 0.01$: compared to the control.

Table S7. PPAR gene expression levels in MSC.

PPAR expression	Relative fold
Control	1.00
Au	0.94
SDF-1α	0.93
Au-Col	1.13
Au-Col-SDF-1α	1.17 *

Note: * $p < 0.05$: compared to the control.
